# Chip-Based Electronic System for Quantum Key Distribution

**DOI:** 10.3390/e26050382

**Published:** 2024-04-29

**Authors:** Siyuan Zhang, Wei Mao, Shaobo Luo, Shihai Sun

**Affiliations:** 1 School of Electronics and Communication Engineering, Sun Yat-sen University, Shenzhen 518107, China; zhangsy39@mail.sysu.edu.cn; 2 Hangzhou Research Institute, Xidian University, No. 8 Qiannong East Road, Hangzhou 710071, China; 3 School of Microelectronics, Southern University of Science and Technology, Shenzhen 518055, China; luos@u.nus.edu

**Keywords:** quantum key distribution, quantum communication, integrated electrical chip

## Abstract

Quantum Key Distribution (QKD) has garnered significant attention due to its unconditional security based on the fundamental principles of quantum mechanics. While QKD has been demonstrated by various groups and commercial QKD products are available, the development of a fully chip-based QKD system, aimed at reducing costs, size, and power consumption, remains a significant technological challenge. Most researchers focus on the optical aspects, leaving the integration of the electronic components largely unexplored. In this paper, we present the design of a fully integrated electrical control chip for QKD applications. The chip, fabricated using 28 nm CMOS technology, comprises five main modules: an ARM processor for digital signal processing, delay cells for timing synchronization, ADC for sampling analog signals from monitors, OPAMP for signal amplification, and DAC for generating the required voltage for phase or intensity modulators. According to the simulations, the minimum delay is 11ps, the open-loop gain of the operational amplifier is 86.2 dB, the sampling rate of the ADC reaches 50 MHz, and the DAC achieves a high rate of 100 MHz. To the best of our knowledge, this marks the first design and evaluation of a fully integrated driver chip for QKD, holding the potential to significantly enhance QKD system performance. Thus, we believe our work could inspire future investigations toward the development of more efficient and reliable QKD systems.

## 1. Introduction

Quantum Key Distribution (QKD) [1] enables two parties to generate an unconditional secret key, the security of which is guaranteed by the fundamental principles of quantum mechanics. Since the proposal of the first QKD protocol, BB84, in 1984, various protocols have been suggested to enhance the performance of QKD in different practical situations, including Continuous-variable QKD [2], Measurement-device-independent QKD [3], twin-field QKD [4], and more. The unconditional security of QKD has been rigorously demonstrated through various methods [1]. Furthermore, the reach of QKD has been extended to hundreds of kilometers [5], and notably, QKD over a distance of 4600 km has been demonstrated by using the Micius satellite [6]. Simultaneously, high-speed QKD with a key rate exceeding 110 Mbps is also available [7].

At the same time, to reduce the size and cost of QKD systems, integrated technology has been introduced to replace bulky optical and electrical devices [8]. In the realm of integrated photonics, ${\mathrm{SiO}}_{2}$-based optical interferometers for QKD were demonstrated in 2004 [9], and numerous other chip-based QKD systems have been implemented [10,11,12,13]. Recently, the first fully integrated chip-to-chip QKD system was demonstrated with an InP transmitter and a silicon oxynitride (${\mathrm{SiO}}_{x}N_{y}$) receiver. The system operates at a clock rate of 1.72 GHz and utilizes the COW protocol over a separation distance of 20 km [14]. In 2020, a cost-effective, mass-producible, chip-based transmitter was proposed [11]. In 2021, a heterogeneous-integrated superconducting silicon photonic chip was reported, which, for the first time, integrated the detector into the chip platform in an MDI QKD system [15]. An integrated QKD photonic chip with 2.5 GHz repetition was also implemented in 2023 [7].

Unlike integrated photonics QKD systems, the integration of electronics in QKD applications has received limited attention. The primary focus of research revolves around the development of driving chips for laser diodes (LDs), modulators, and detectors. Various pioneering efforts have been documented. In 2019, Zhu proposed a laser modulator driver based on 130 nm CMOS technology that employs active feedback [16]. In 2020, Wang introduced a pre-driver chip, utilizing a 130 nm CMOS process for QKD applications [8]. This integrated Application-Specific Integrated Circuit (ASIC) electronic system serves as the driving signal for the optical device at the sender. In 2021, with 65 nm CMOS technology, Jiang designed an active quench and reset electronic chip for the single-photon avalanche detector (SPAD) [17]. Most recently, using integrated silicon photonics and electronics technology, Zhu devised an integrated optoelectronic QKD transmitter in 2022 [18]. A successful QKD experiment was then demonstrated with a repetition frequency of 312.5 MHz and a key rate of 42.7 kbps for 100 km of fiber and 295.5 bps for 40 dB emulated loss. Existing research on the integration of QKD electronic control systems has not achieved the monolithic integration of the entire system onto a single chip.

In this paper, we propose a new design of an integrated electronic chip for the application of QKD. Based on 28 nm CMOS technology, our chip generates driving signals for lasers and modulators, concurrently monitoring optical intensity in real time. Simultaneously, a programmable clock delay module is designed to ensure accurate loading of all driving signals into the optical devices. Furthermore, our method for generating the driving signals differs from Zhu’s approach; they employ a voltage-controlled delay chain, whereas we utilize a digital-to-analog converter (DAC) for signal generation. Our chip includes five modules: an ARM for real-time data processing; a delay cell, introducing a delay to the clock signal; an analog-to-digital converter (ADC), monitoring laser output light intensity; an operational amplifier (OPAMP), amplifying the signal before ADC sampling; and a DAC, converting digital signals into analog signals. Such an integrated design reduces power consumption, minimizes area overhead, and achieves greater miniaturization and lower cost for the overall system.

## 2. Optical and Electrical System of QKD

### 2.1. Optical Structure of QKD System

**Figure 1:**
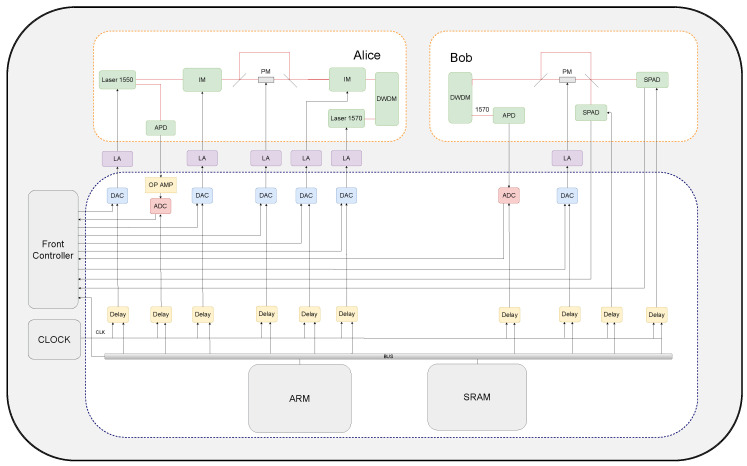
A typical QKD system with optical and electrical devices. IM (PM) is the optical intensity (phase) modulator; DWDM stands for Dense Wavelength Division Multiplexing; APD is the avalanche photodiode; SPAD is the single photon detector; LA is the limiting amplifier; DAC is the digital-to-analog converter; OPAMP is the operational amplifier; ADC is the analog-to-digital converter; Delay is the programmable signal delay module; ARM is the processor architecture; SRAM is the static random-access memory. In our design, the LA is not included in the integrated electrical chip since it is a high power module.

Figure 1 shows a typical QKD system with phase or time-bin encoding (the same functional modules are also used for polarization encoding). The optical system contains four parts: (1) A laser diode (LD) with 1550 nm generates quantum optical pulses, and a laser diode with a wavelength of 1570 nm is used to transmit Alice’s clock signal. The quantum signal has a typical width of about 30∼50 ps and repetition of 50 MHz∼1.25 GHz. And the repetition of the classical clock synchronization is about 10 MHz. (2) The phase and intensity modulators (IM and PM in Figure 1) are used to change the intensity and phase of the quantum optical pulses; then, different quantum states can be encoded and decoded. The modulators have a typical half-wave voltage of 3∼5 V. (3) The single-photon detectors (SPD) are used to detect the single-photon quantum state. (4) The photonic diode measures classical optical signals to ensure that the QKD system operates within correct parameters. For example, it can monitor the intensity of the quantum signal laser and detect the classical clock synchronization signal to ensure Alice and Bob share the same clock.

### 2.2. Electrical Structure of QKD System

Figure 1 also shows the structure of our integrated electrical chip. To drive the optical system of QKD, the designed electrical chip includes five basic modules: ARM, DELAY, ADC, OPAMP, and DAC. Note that the front controller used for data processing and system control is not included in our chip. Additionally, the LA is not integrated since it is a high-power module.

For the driving of LD, an electrical signal with adjustable width and amplitude is used to generate the optical pulse with the required width and intensity [19]. This function is implemented with a DAC combined with an LA [20].The front controller outputs the required data to the DAC, and the clock of DAC is controlled by the ARM to ensure that the optical pulses are generated in a suitable time slot. The chip is designed with a driving signal frequency of 50 MHz, adjustable up to 500 mV and with a step size of 3.125 mV, to drive different types of LD.

To implement the encoding and decoding of quantum states, the IM and PM necessitate drive signals with varying voltages [21]. The DAC generates a low-voltage signal, subsequently amplified by the LA. In our design, the DAC’s maximal output voltage is ±0.5 V. Therefore, we employ an LA with a gain of 10–13 dB to ensure that a phase shift of about 2π can be imparted to the modulators (where the half-wave voltage of PM and IM ranges from 3 to 5 V). Simultaneously, the DAC’s clock is regulated by the ARM and delayed to ensure that the drive signal is applied to the modulators only when the optical pulse passes through them.

Another vital function of our chip is to sample and process both analog and digital signals, ensuring the QKD system operates within the correct parameter range. Specifically, through the use of an OPAMP and an ADC, we stabilize the output optical power of the LDs. The LD optical power’s fluctuations can otherwise diminish the QKD key rate and introduce potential security vulnerabilities. Simultaneously, the trigger signal for the SPADs is controlled by ARM and a delay mechanism to ensure that SPADs only operate when optical pulses are received. The chip also directly samples the output digital signal of SPADs for subsequent processing, which is conducted as post-processing in QKD. It is important to note that the front controller handles this post-processing, and it is not integrated into the chip.

## 3. Electronic Chip Design

The chip is designed using 28 nm CMOS technology and consists primarily of five modules: ARM, DELAY, ADC, OPAMP, and DAC.

### 3.1. ARM

The processor adopted in our chip is the microprocessor of the Cortex-M series that the ARM company provides, which has the advantages of a small size, low power consumption, low cost, high performance, etc., and is widely used in embedded systems. The processor, which employs the Harvard architecture, features separate instruction and data buses, enabling simultaneous instruction fetching and data access. The ARM processor is used to provide instruction signals to the delayer, provide data to the DAC, and collect and process the data collected by the ADC and SPAD.

### 3.2. DELAY

**Figure 2:**
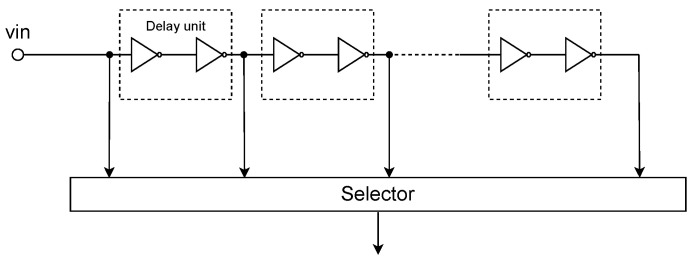
Schematic diagram of the DELAY. The number of delay units controlled by ARM.

As shown in Figure 2, the designed circuit includes multiple series-connected inverters, and each inverter introduces a certain delay [22]. Two inverters together form a delay unit to prevent signal inversion. By increasing or decreasing the number of inverters, the overall delay can be controlled by the ARM to intervene with the quantity of inverters. This method results in the advantage of lower jitter in the designed delay. With each delay unit introducing a delay of $T_{LSB}$ and a total of *N* delay units being traversed, the overall delay is $NT_{LSB}$.

### 3.3. ADC

**Figure 3:**
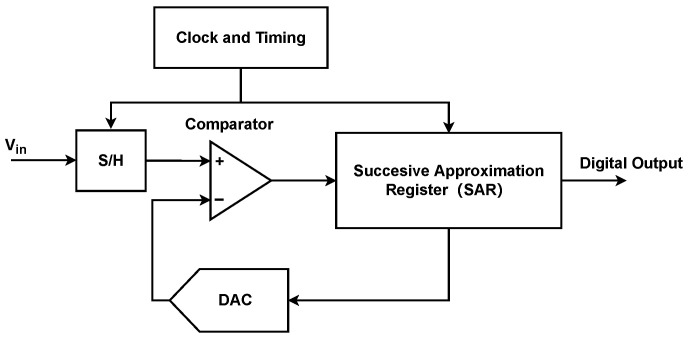
Schematic diagram of the ADC.

The Successive Approximation Register (SAR) ADC [23] is adopted in the design of ADC, which has the advantages of low power consumption, small area, and simple structure. As shown in Figure 3, it includes a sample and hold circuit (labeled as S/H in the figure), a DAC, a comparator, and a digital logic part of SAR. In the SAR ADC, the sampled signal is held as $V_{in}$, then $V_{DAC}$ and $V_{in}$ are constantly compared [24]. The value of $V_{DAC}$ is changed according to the result of the comparator, which is stored in a register as a binary number before being output.

### 3.4. OPAMP

**Figure 4:**
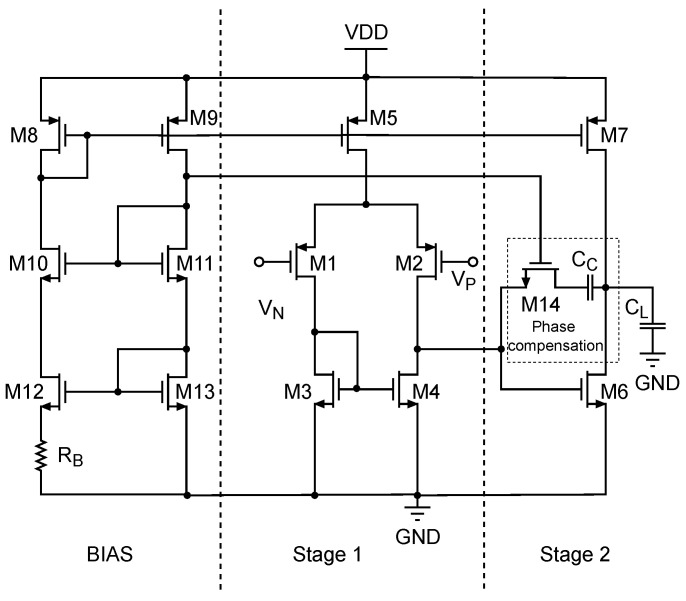
Circuit schematic of a two-Stage Miller-compensated operational amplifier. The phase compensation circuit consists of M14 and $C_{c}$.

In order to sample small voltage signals from APD and other system monitors, amplification is necessary. Therefore, operational amplifiers are integrated into the chip to provide the required amplification. The operational amplifiers used in this text are two-stage Miller OPAMPs, as shown in Figure 4. It mainly consists of four parts [25]: the first-stage input amplification circuit, the second-stage amplification circuit, the biasing circuit, and the phase compensation circuit. Miller capacitors are directly connected between the two stages of amplification, utilizing the Miller effect to improve the frequency characteristics of the circuit by shifting the positions of adjacent poles.

### 3.5. DAC

**Figure 5:**
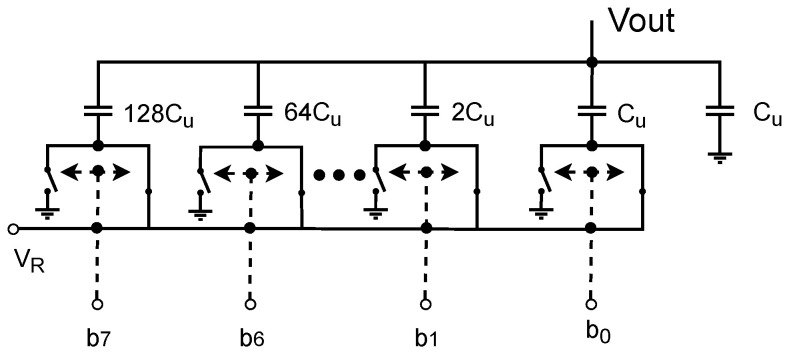
Circuit diagram of the weight capacitor array of DAC. $C_{u}$ represents the base capacitance value.

For the design of DAC, the weight capacitor array structure is adopted. As shown in Figure 5, it divides the total charge of the capacitor array into binary partitions by attenuating the reference voltage with capacitors [26]. This type of DAC only consumes power when the capacitor is charging or discharging, which can greatly reduce power consumption compared to other DACs.

## 4. Test of the Electronic Chip

Based on the design given above, we simulate the performance of our designed chip. The test results show that the performance of each module meets the requirements of QKD. Additionally, the exceptionally low power consumption and compact size significantly reduce the overall power and space requirements of the chip, offering a practical solution for the miniaturization of QKD devices.

### 4.1. DELAY

We continuously vary the number of inverters in the connected circuit, and the simulation results show that the delay of each delay cell is 11 ps, with a delay fluctuation range of 1 ps. The detail results are listed in Table 1.

### 4.2. ADC

We input a square wave signal and test the output of the ADC. According to the requirements, two ADCs with different sampling rates are designed, namely, a low-speed ADC with a sampling rate of 100 kHz and a high-speed ADC with a sampling rate of 50 MHz. The performance of both ADCs is listed in Table 2.

### 4.3. OPAMP

As shown in Table 3, the open-loop gain of the operational amplifier is 86.2 dB, the bandwidth is 100 MHz, the settling time from 10% to 90% is 0.5 ns, and the output amplitude is +0.5V to −0.5V.

### 4.4. DAC

We convert a square wave signal into a digital signal and input it to the DAC, thereby testing the performance indicators of the DAC. According to the requirements, two DACs with different sampling rates are designed, namely, a low-speed DAC with a sampling rate of 100 kHz and a high-speed DAC with a sampling rate of 100 MHz. The performance of both DACs is listed in Table 4.

## 5. Discussion

QKD has the potential application of guaranteeing the unconditional security of data. One of the main trends of QKD is that it integrates all photonics and electronic devices together. Then, the performance of QKD in size, cost, and power consumption could be further reduced. In this paper, we designed a chip-based electrical system for quantum key distribution in a 28 nm CMOS process. In order to ensure that the QKD system can be worked correctly, the designed electronic chip provides the driving signals for all optical devices and samples the corresponding digital and analog signals. Such an integrated design reduces power consumption, minimizes the area overhead, and achieves a greater miniaturization and lower cost of the overall system. This is of great significance for practical applications in terms of portability and energy efficiency. It is worth noting that our design has high flexibility. Subsequent designs can be tailored specifically for other QKD protocols without the need of modifying the overall architecture. We simply need to adjust the internal components, such as modulators and detectors, according to the requirements of the new protocol, allowing for rapid adaptation to different QKD application scenarios. This modular and customizable design makes our chip highly versatile. Thus, we believe that our design provides a very competitive way to implement QKD with a higher performance. However, the challenge of how to achieve higher-speed systems with the control chip remains. In the future, we plan to further enhance the performance, reduce costs, and expand the application scope of the chip.

## Figures and Tables

**Table 1:** Performance specifications of delay.

Parameter	Value
Minimum Delay	11 ps
Delay Step	10 bits
Time Jitter	1 ps
Maximum Frequency	500 MHz
Settling Time From 20% to 80%	2.5 ps

**Table 2:** Performance specifications of ADC.

Parameter	Low-Speed ADC	High-Speed ADC
Sampling Rate	100 kHz	50 MHz
Bit Width	8 bits	8 bits
Power Consumption	6.66 μW	79 μW
Supply Voltage	0.8 V	0.8 V
Analog Input Range	+0.5 V/−0.5 V	+0.5 V/−0.5 V
Settling Time From 10% to 90%	50 ps	11 ps

**Table 3:** Performance specifications of operational amplifiers.

Parameter	Value
Open-loop Gain	86.2 dB
Bandwidth	100 MHz
Settling Time From 10% to 90%	0.5 ns
Output Amplitude	+0.5 V/−0.5 V

**Table 4:** Performance specifications of DAC.

Parameter	Low-Speed DAC	High-Speed DAC
Sampling Rate	100 kHz	100 MHz
Bit Width	8 bits	8 bits
Power Consumption	0.13 μW	0.73 μW
Supply Voltage	0.8 V	0.8 V
Reference Voltage	0.8 V	0.8 V
Output Amplitude	+0.5 V/−0.5 V	+0.5 V/−0.5 V
Settling Time to 0.1%	65 ns	0.75 ns
Settling Time From 10% to 90%	3250 ps	32 ps
Output Noise	27.9 nV/sqrt (Hz)	3.8 nV/sqrt (Hz)

## Data Availability

No new data were created or analyzed in this study. Data sharing is not applicable to this article.
